# Impact of Ciprofloxacin and Chloramphenicol on the Lipid Bilayer of *Staphylococcus aureus*: Changes in Membrane Potential

**DOI:** 10.1155/2013/276524

**Published:** 2013-05-23

**Authors:** Paulina L. Páez, María C. Becerra, Inés Albesa

**Affiliations:** Departamento de Farmacia, Facultad de Ciencias Químicas, Universidad Nacional de Córdoba, Haya de la Torre y Medina Allende, Ciudad Universitaria, IMBIV-CONICET, 5000 Córdoba, Argentina

## Abstract

The present study was undertaken to explore the interaction of ciprofloxacin and chloramphenicol with bacterial membranes in a sensitive and in a resistant strains of *Staphylococcus aureus* by using 1-anilino-8-naphthalene sulfonate (ANS). The binding of this probe to the cell membrane depends on the surface potential, which modulates the binding constant to the membrane. We observed that these antibiotics interacted with the bilayer, thus affecting the electrostatic surface potential. Alterations caused by antibiotics on the surface of the bacteria were accompanied by a reduction in the number of binding sites and an increase in the ANS dissociation constant in the sensitive strain, whereas in the ciprofloxacin-resistant strain no significant changes were detected. The changes seen in the electrostatic surface potential generated in the membrane of *S. aureus* by the antibiotics provide new aspects concerning their action on the bacterial cell.

## 1. Introduction

The plasmatic membrane is a chemoosmotic barrier that provides an interface between the organism and the environment. This bilayer presents an electrochemical potential (negative in the interior) which plays a basic role in the control of the exchange of solutes. Disturbances in the membrane potential can provide a rapid and sensitive indication of those stimuli that lead to physiological functionally important changes with respect to bacterial viability [1].

Fluorescent molecules have been extensively used as probes of biological membranes. These hydrophobic and amphiphilic probes are associated with membranes when added to cells or artificial systems, and their resultant fluorescence properties can be used to monitor a variety of membrane characteristics. In general, the addition of effectors results in the deenergization of cells, which leads to increased fluorescence from the probes present in the cell suspension, such as negatively charged 8-anilino-1-naphthalenesulfonate (ANS) [2]. 

ANS binding and fluorescence strongly respond to modulation of the surface potential, with the energy-dependent quenching being largely due to the generation of ΔΨ and being accounted for by the movement of the anion across the membrane and from intramembrane sites in response to membrane potential [3]. 

It has been demonstrated that the determination of the membrane potential based on fluorochromes provides a useful and sensitive approximation for the monitoring of the cellular stresses in bacteria [4–6], since both oxidative and nitrosative stress are able to depolarize the plasmatic membrane [7].

The effect of the oxidative stress generated by reactive oxygen species (ROS) has been described as one of the most important sources of metabolic disturbance and the cellular damage. These agents are involved in the first important changes in the plasmatic membrane, and consequently at the beginning of cellular death [8–10].

Bacterial gyrase inhibitors, including synthetic quinolone antibiotics, induce a breakdown in iron regulatory dynamics, which promotes the formation of the ROS that contribute to cell death [11].

Bactericidal antibiotic killing mechanisms are currently attributed to the class of specific drug-target interactions. However, the understanding of many of the bacterial responses that occur as a consequence of the primary drug-target interaction remains incomplete. It is known that oxidative stress in bacteria can be caused by exogenous agents that originate toxic effects, and our previous studies have shown that ciprofloxacin (CIP) and chloramphenicol (CMP), among others, can stimulate the induction of ROS in different bacterial species [12–16].

The aim of the present study was to explore the effects of clinically used antibiotics such as CIP and CMP on the lipid surface and to estimate the variation in the membrane potential in *Staphylococcus aureus *strains.

## 2. Materials and Methods

### 2.1. Susceptibility Determination

The antimicrobial activities of CIP and CMP were evaluated in two strains, one standard strain *S. aureus* ATCC 29213 and other clinical strain *S. aureus* by using the standard tube dilution method following the indications of the Clinical and Laboratory Standards Institute [17]. The strains were maintained by culture in trypticase soy broth (TSB) for 24 h at 37°C, and the minimum inhibitory concentration (MIC) was determined by using the standard tube dilution method. Cultures of 24 h in Mueller-Hinton medium were diluted to 10^6^ CFU/mL, incubated for 10 min at 37°C, and then the antibiotics were added at different concentrations (0.125 *μ*g/mL–512 *μ*g/mL). Bacterial growth was observed at 24 h of incubation. MIC was determined as the lowest antibiotic concentrations at which growth was completely inhibited after overnight incubation of the tubes at 37°C. MICs were determined three times and the median values are taken.

### 2.2. ANS Binding Studies

Overnight cultures of *S. aureus* ATCC 29213 and clinical strain *S. aureus* were prepared in trypticase soy broth. Suspensions were centrifuged, and the pellets were resuspended in saline phosphate buffer (PBS) pH 7.4 at an optical density of 0.4 at 600 nm. Then, 50 *μ*L of these suspensions was incubated with 256 *μ*g/mL of CIP, 4 *μ*g/mL of CMP, or without antibiotic (control) in a total volume of 1 mL in PBS.

The suspensions were centrifuged, and 1 mL of Triton 1% V/V in NaCl 10% was added to the pellet. Then, 20 *μ*L of ANS 60 *μ*M was added to 50 *μ*L of bacterial suspensions and PBS to a total volume of 3 mL. The assay mixture for the standard curve consisted of 1 mL of bacterial suspensions and different concentrations of ANS, ranging from 0 to 120 *μ*M. The structural changes on the membrane potential were studied by using L-anilino naphthalene-8-sulphonate as the fluorescent probe by the method of Verma et al. [18] and Robertson and Rottenberg [19]. The fluorescence emission was recorded on a Spectrofluorometer PTI (Photon Technology International) Model Quanta Master 2 QM2, with phosphorescence lifetime measurements taken at excitation and emission wavelengths of 360 nm and 516 nm, respectively. These experiments were performed at room temperature (23°C). 

### 2.3. Measurement of *K*_*d*_ and *n* from ANS by Fluorescence Emission in Bacteria

The approach used to determine the dissociation constant (*K*_*d*_) and the number of binding sites (*n*) from the fluorescence yield was as previously reported by Verma et al. [18] and Robertson and Rottenberg [19]. The fluorescence developed is recorded, and the data is plotted as the reciprocal of the fluorescence signal (arbitrary units) versus the reciprocal of the concentration of ANS. This produces a straight line whose extrapolation with the ordinate gives the reciprocal of the limiting fluorescence of ANS (*F*_max⁡_). The number of binding sites for ANS was calculated by plotting the bound ANS per mg protein/free ANS versus bound ANS per mg protein. 

### 2.4. Statistical Analysis

The assays were carried out at least in triplicate. Data were expressed as mean ± SD and analyzed by the Student's *t*-test. *P* < 0.05 was used as the level of statistical significance.

## 3. Results and Discussion

*S. aureus* ATCC 29213 exhibited sensitivity to CIP and CMP, with MICs of 0.5 *μ*g/mL for CIP and 1 *μ*g/mL for CMP. In addition, the clinical strain *S. aureus* MICs obtained were 32 *μ*g/mL for CIP and 8 *μ*g/mL for CMP; according to these results, the strain was resistant to CIP but sensitive to CMP. The fluorescence emission of ANS at 516 nm in the presence of 256 *μ*g/mL of CIP, 4 *μ*g/mL of CMP or in the absence of antibiotic was determined with *S. aureus* ATCC 29213 and clinical strain *S. aureus*.

Data were plotted as the reciprocal of the fluorescence signal (1/F) versus the reciprocal of the concentration of free ANS (1/ANS), as the reciprocal of the intercept gives the limit of the ANS fluorescence (*F*_max⁡_), a parameter related to the maximum concentration of bound ANS. From the slope, the *K*_*d*_ was obtained from which the affinity of the fluorescent probe for binding sites on the bilayer could be inferred.

The values of bound ANS and free ANS were calculated from (1), where

(1) $$\begin{array}{c} \text{ANS}\;\text{bound}\;\left(\text{nmol}\right)=\frac{1/F_{\max}}{1/F}\times 100,\\ {\text{ANS}}_{\text{free}}\left(\text{nmol}\right)={\text{ANS}}_{\text{total}}-{\text{ANS}}_{\text{bound}}. \end{array}$$

The surface potential (Ψ) was calculated according to (2):

(2) $$\begin{array}{c} \Psi =59\;\log\left(F/F_{o}\right), \end{array}$$

where *F* corresponds to the fluorescence in the presence of each antibiotic and *F*_*o*_ corresponds to the fluorescence obtained when Ψ = 0.

The change in membrane potential (ΔΨ) in the presence of antibiotic with respect to the control without antibiotic was obtained from the difference between Ψ with and without antibiotic (3):

(3) $$\begin{array}{c} \Delta \Psi =\Psi -\Psi_{\text{control}}. \end{array}$$

The number of binding sites for ANS was calculated by using (4), in which the intercept represents the value of the reciprocal of *q* (a constant of proportionality), with the value of *n* being obtained from the slope (*K*_*d*_/*qn*):

(4) $$\begin{array}{c} \frac{{\text{ANS}}_{\text{free}}}{F}=\frac{K_{d}}{qn}\times \frac{1}{\left[\text{bacteria}\right]}+\frac{1}{q}. \end{array}$$

The same procedure was performed to determine the number of binding sites for ANS when the bacterial suspensions were incubated with the antibiotics studied, where a decrease in the number of binding sites for ANS with antibiotic would suggest changes in the cell surface.

Any alteration in the binding of ANS to the membrane of *S. aureus* in the presence of antibiotics was tested by a decrease in the number of binding sites for ANS compared with control. When ANS bounds to phospholipids, the fluorescence intensity increased with high concentrations of ANS. However, in the presence of CMP or CIP, the ANS fluorescence decreased, with this behavior reflecting competition between CMP and the ANS binding site that might have been located at the interface of the membrane. By comparing the values of *K*_*d*_, we were able to infer that the affinity of the probe sites of the bilayer was affected by the two antibiotics. Moreover, differences in the values of the electric potentials indicated alterations in the bacterial membranes.

**Figure 1:**
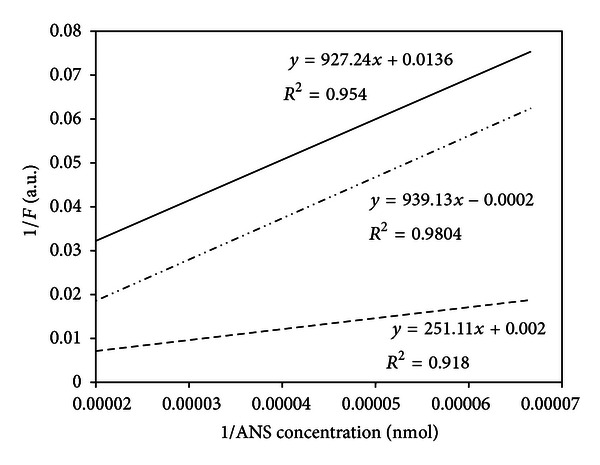
Scatchard plots of ANS interaction with *S. aureus* ATCC 29213 control (- - -), treated with ciprofloxacin (—) and treated with CMP (-··-).

The fluorescence of ANS emission was determined at 516 nm in the presence of CIP and CMP and in the absence of antibiotic. The data were plotted as the reciprocal of the fluorescence signal (1/*F*) versus the reciprocal of the concentration of ANS (1/ANS) (Figure 1). There was a linear relationship between fluorescence and the inverse of the concentration of bound ANS, revealing a change in the affinity of the membrane in the presence of antibiotic with respect to control.

Table 1 shows the parameters obtained with CMP and CIP for the surface potential in the absence or presence of the antibiotics in the *S. aureus *ATCC 29213 and clinical strain *S. aureus*. 

Whereas the value of *F*_max⁡_ was reduced twofold and the number of binding sites decreased elevenfold, the *K*_*d*_ value was increased fourfold, compared to the control without antibiotic in *S. aureus* ATCC 29213 (sensitive to CIP). In clinical strain *S. aureus* (resistant to CIP) there were no significant changes in *F*_max⁡_, number of binding sites, or the *K*_*d*_ value. The baseline value of Ψ in the sensitive strain was −306 mV, while in the resistant strain this was −287 mV. The ΔΨ was almost seventimes higher in the sensitive strain (100 mV) than in the resistant one (15 mV). CIP, a bactericidal antibiotic, reduced this value by 33% in the sensitive strain, while the reduction in the resistant one was only 5%. Finally, CMP, a bacteriostatic antibiotic, increased the membrane potential by about 11% in the sensitive strain. 

The membrane potential is an important parameter that controls various cellular processes. It is a sensitive indicator of energy status and cell viability, with membrane depolarization leading to excessive production of ROS which is an indication of an advance in cellular dysfunction and precedes many other signs of cellular injury. A reduction in the potential also provides information about the feasibility of transferring an electron “*in vivo.*” In addition, the catalytic production of oxidative stress from the redox cycle is a possible mode of action of antibiotics, because it could indicate interference with the electron transport chain [20, 21].

The changes in the electric potential obtained in the present work showed alterations in the bacterial membrane of *S. aureus* in the presence of CMP and CIP. Furthermore, changes caused by antibiotics on the surface of the bacteria were demonstrated by a reduction in the number of binding sites of the fluorescent probe and an increase in the ANS dissociation constant.

Montero et al. established that CIP interacts with neutral and charged membranes at the surface level (headgroup region). They also postulated that this could be part of the mechanism of entry of the 6-fluoroquinolones through the cytoplasmic membrane [3]. Ciprofloxacin is a widely used antimicrobial agent against Gram positive and Gram negative, but there are conflicting reports about the effect of CIP on the bacterial membrane [22, 23]. 

There are previous results on *S. aureus*, with liposomes of *E. coli* showing interaction of ANS with a lipid bilayer and in the presence of 6-FQs as a result of a reduction in the maximum concentration of the ANS bound to liposomes [24–27]. However, there are no comparisons reported between sensitive and resistant strains in the presence of antibiotics.

The present results demonstrated that the strains had a particular behavior in the presence of each antibiotic, an effect that was manifested by the differences obtained in the bacterial membrane potential. Moreover, on comparing the sensitive strain with the resistant one, a higher alteration in the membrane potential was observed in sensitive bacterium that was associated with the effect of the antibiotic [20, 21].

## 4. Conclusions

In our previous reports, we demonstrated that CIP and CMP induce oxidative stress in* S. aureus* strains, with sensitive clinical strains producing higher ROS levels than resistant ones [12–15]. The present work shows that these antibiotics had an impact on the lipid bilayer, leading to significant membrane potential changes in *S. aureus* sensitive to ciprofloxacin. These observations can be added to the mechanism of action previously described for the antibiotics investigated, with the changes generated in the lipid bilayers of *S. aureus *contributing new aspects about the action of these antibiotics on the bacterial cell.

## Figures and Tables

**Table 1:** Parameters obtained from the ANS binding studies in *S*.  *aureus* ATCC 29213 and clinical strain *S.  aureus*.

	*F*_max⁡_	*K*_*d*_	*n *	Ψ (mV)	ΔΨ (mV)
*S. aureus* ATCC 29213 sensitive to CIP and CMP					
Control without antibiotic	143	251	603028	−306	—
Ciprofloxacin 256 *μ*g/mL	70	927	52983	−406	−100
Chloramphenicol 4 *μ*g/mL	62	846	48332	−331	−25

Clinical strain *S. aureus* resistant to CIP					

Control without antibiotic	77	902	45110	−287	—
Ciprofloxacin 256 *μ*g/mL	77	937	46865	−302	−15

*F*_max⁡_ is the fluorescence intensity (related to the maximum concentration of bound ANS), *n* is the number of binding sites of ANS to the membrane, *K*_*d*_ is the dissociation constant, Ψ is the potential at the surface of the membrane and ΔΨ is the change in surface potential of the membrane in the control without antibiotic.
